# Chromosome-Scale Genome Analysis Reveals Locus-Specific Disruption of the Citrinin-Associated Region in a Furu-Derived *Monascus ruber* Strain BC20

**DOI:** 10.3390/foods15173091

**Published:** 2026-08-31

**Authors:** Huanchang Zhang, Danqi Wang, Jin Han, Fenghua Zhang, Yiru Liu, Rui Liu, Miya Su, Su Yao, Zhenmin Liu

**Affiliations:** 1 State Key Laboratory of Dairy Biotechnology, Key Laboratory of Functional Dairy Products Processing, Ministry of Agriculture and Rural Affairs, Shanghai Engineering Research Center of Dairy Biotechnology, Dairy Research Institute, Bright Dairy and Food Co., Ltd., Shanghai, 201103, China; 2 Fudan Microbiome Center, School of Life Sciences, Fudan University, No. 2005 Songhu Road, Yangpu District, Shanghai 200438, China; 3 China Center of Industrial Culture Collection, China National Research Institute of Food & Fermentation Industries Corporation Limited, Beijing, 100015, China

**Keywords:** comparative genomics, biosynthetic gene cluster, synteny analysis, transposable elements, food fermentation

## Abstract

*Monascus* species are widely used in traditional fermented foods for pigment and flavor formation, but citrinin contamination remains a major safety concern that limits broader food applications. Therefore, this study aimed to evaluate the citrinin risk of a furu-derived *Monascus ruber* strain, BC20, by integrating phenotypic screening across food-relevant matrices with genome-resolved analysis. After 14 days of cultivation across eight matrices, including fungal media as well as dairy-, cereal-, and bran-based substrates, citrinin was not detected by immunoaffinity cleanup combined with HPLC–FLD (LOD, 4 μg/kg; LOQ, 12 μg/kg). To investigate the genetic basis of this phenotype, we generated a chromosome-scale genome assembly for BC20 and conducted comparative analyses across a total of 19 *Monascus* genomes. ANI analysis and phylogenomic inference consistently placed BC20 within the *ruber–pilosus* clade. Comparative synteny analysis showed that the citrinin-associated locus in BC20 no longer retained an intact cluster configuration but instead exhibited a remnant-locus architecture, and similar patterns were also observed in several related genomes from the same clade. By contrast, the monacolin K (*mk*) locus remained syntenically conserved in BC20, supporting locus-specific structural disturbance rather than assembly-derived pseudo-absence. Additionally, its antifungal susceptibility was determined. Overall, BC20 represents a *M. ruber* candidate strain with undetectable citrinin, and this study provides a practical analytical framework for citrinin risk screening in food-related *Monascus* isolates.

## 1. Introduction

The genus *Monascus* is one of the most important traditional fungal groups used in food fermentation [1]. It has long been applied in East Asian fermented foods for pigment formation, flavor development, and the production of secondary metabolites associated with fermentation quality [2]. In addition to its long-established use in traditional products such as red yeast rice and fermented tofu, *Monascus* is increasingly being considered for application in a wider range of cereal-based substrates [3] and dairy-related systems [4]. In particular, its application in cheese ripening provides a relevant basis for evaluating food-associated *Monascus* strains in dairy matrices. In furu fermentation, *Monascus* may constitute an important component of the fungal community, particularly during post-fermentation. One longitudinal study reported that *Aspergillus* and *Monascus* together accounted for approximately 95% of fungal sequences during 7–84 days of sufu post-fermentation, while another study identified *Monascus* as the most abundant fungal genus throughout the post-fermentation period [5]. Its substantial occurrence in the furu fungal community also makes fermented tofu a relevant source for the isolation of naturally occurring food-associated *Monascus* strains. For the food industry, the development of strains with a clear genetic background, controllable fermentation behavior, and verified safety is essential.

However, the broader application of *Monascus* has long been limited by the risk of citrinin contamination [6]. Citrinin is a polyketide mycotoxin that is frequently detected in some *Monascus* fermentation products, making safety control and regulatory compliance major obstacles to industrial application [7]. Regulatory limits for citrinin are product-specific; for example, China sets a maximum level of 0.04 mg/kg for Monascus red (GB 1886.181-2016) [7], whereas the European Union sets a maximum level of 100 μg/kg for food supplements based on rice fermented with *M. purpureus* (Regulation (EU) 2023/915). Previous studies have shown that citrinin biosynthesis is mainly governed by a clustered biosynthetic gene cluster centered on key genes such as *ctnA*, together with multiple modifying and regulatory genes [8]. Although toxin production can be reduced by process optimization or genetic engineering, transgenic approaches often face regulatory constraints and low consumer acceptance in food applications. Therefore, the isolation of naturally stable citrinin-negative *Monascus* strains from environmental sources, supported by reproducible genetic and experimental evidence, is a more practical strategy.

Comparative genomic studies have shown that the citrinin biosynthetic region varies among *Monascus* species and strains, with differences in gene content and cluster integrity reported for *M. purpureus*, *M. ruber*, and *M. pilosus* [9,10,11,12]. These studies indicate that citrinin-producing potential is closely associated with the structural state of the corresponding biosynthetic locus. However, for naturally isolated citrinin-negative strains, whether this phenotype remains stable across food-relevant conditions and whether it reflects structural disruption of the locus rather than condition-dependent silencing remain insufficiently characterized.

At least two scenarios are possible. One is structural deletion or disruption within the citrinin biosynthetic gene cluster, which physically interrupts the pathway. The other is that the cluster remains intact but is conditionally silenced or downregulated, leading to non-detection under limited test conditions. These two scenarios have different implications for industrial use. Structural disruption is generally more stable, whereas conditional silencing requires broader validation across environmental conditions and gene expression states [13]. Based on these considerations, we hypothesized that a consistently non-detectable citrinin phenotype across food-relevant conditions would be associated with structural disruption of the citrinin-associated locus rather than condition-dependent silencing.

Transposable elements are common drivers of structural variation in fungal genomes and may contribute to the gain or loss of secondary metabolite gene clusters through insertion, deletion, or rearrangement [14]. However, without breakpoint-level validation, the co-localization of transposable elements with the altered locus should be interpreted as a structural association rather than direct evidence of a causal mechanism.

To test this hypothesis, we examined the citrinin phenotype of the furu-derived *Monascus ruber* strain BC20 across eight culture systems, including standard fungal media and food-related matrices. We then generated a chromosome-scale genome assembly and investigated the status of the citrinin-associated locus in an intragenus comparative framework. The *mk* locus was used as a control to exclude pseudo-absence, while antifungal susceptibility profiling was included as additional strain-characterization information. Finally, we evaluated the fermentation-related genetic potential of BC20 through functional annotation. Together, these analyses were used to assess the citrinin risk of BC20 and to determine whether its phenotype was supported by locus-level genomic evidence.

## 2. Materials and Methods

### 2.1. Strain Isolation and Identification

Fungal colonies were isolated from farmhouse fermented tofu (furu) samples collected in Wuhan, China. Following repeated streaking and purification on malt extract agar (MEA) and potato dextrose agar (PDA) plates, a total of 10 *Monascus* isolates were obtained. BC20 was selected from these isolates for further characterization. Colony morphology was observed on MEA plates after incubation at 30 °C for 7 days.

Genomic DNA was extracted using a commercial kit (Tiangen BiotechBeijing, China). Molecular identification was performed using ITS rDNA and β-tubulin (*BenA*) sequences. The ITS region was amplified using the ITS1/ITS4 primer pair, and the *BenA* gene was amplified using Bt2a/Bt2b primers. Each 25 μL PCR mixture contained 12.5 μL of 2× Master Mix, 0.5 μL of each primer (10 μM), 1 μL of template DNA (10–50 ng), and ddH_2_O to volume. PCR conditions were as follows: initial denaturation at 94 °C for 5 min; 35 cycles of 94 °C for 30 s, 55 °C for 45 s for ITS or 58 °C for 60 s for *BenA*, and 72 °C for 60 s; followed by a final extension at 72 °C for 10 min. Purified amplicons were subjected to Sanger sequencing by the China National Research Institute of Food & Fermentation Industries (Beijing, China).

The obtained sequences were searched against the NCBI GenBank database using BLASTn(BLAST+ v2.17.0) to identify closely related species [15]. Multiple sequence alignments were performed in MEGA 5.0, and phylogenetic trees were constructed using the neighbor-joining method with 1000 bootstrap replicates [16]. To reduce rooting bias caused by differences in outgroup sequence availability among genes, the ITS tree was rooted with *Amorphotheca resinae* ATCC 200942 (AF393726), whereas the *BenA* tree was rooted with *Scytalidium thermophilum* CBS 622.91 (KF958000).

### 2.2. Inoculum Preparation, Matrices, and Fermentation Conditions

The standardized spore suspension was inoculated into eight cultivation systems at 2% (*v*/*v* for liquid media) or 2% (*v*/*w* for solid media). These included basal fungal media and simulated food-related matrices. The fungal media were yeast extract sucrose (YES) broth and Czapek-Dox broth supplemented with yeast extract and 20% sucrose (CY20). Cereal-based matrices included rice-corn steep (RC) medium, rice medium (RM), corn medium (CM), wheat bran medium (WBM), and a mixed rice-corn-wheat bran medium (RCW). The dairy matrix consisted of commercially sterilized milk (FM; Bright Dairy). Liquid cultures were incubated at 30 °C and 120 rpm, whereas solid cultures were incubated statically at 30 °C. All cultures were maintained for 14 days with three independent biological replicates (*n* = 3) per condition. The initial and post-fermentation pH values were determined for all cultivation systems, and the initial water activity (aw) was measured for the solid substrates. At the end of the 14-day cultivation period, the cultures from each system were collected for subsequent citrinin extraction and analysis. Detailed compositions, sterilization procedures, and incubation parameters are listed in Supplementary Table S1.

### 2.3. Citrinin Extraction and HPLC–FLD Analysis

After fermentation, 2.0 g of solid sample or 2.0 mL of liquid sample was extracted with 10 mL of 70% (*v*/*v*) aqueous methanol for 20 min at room temperature. After centrifugation at 8000× *g* for 10 min at 4 °C, the fermentation extracts was purified using a citrinin immunoaffinity column according to the manufacturer’s instructions. The methanolic eluate was dried under nitrogen at 50 °C, reconstituted in the initial mobile phase, filtered through a 0.22 μm organic membrane, and analyzed. All tested matrices were processed using the same extraction and cleanup procedure for qualitative screening.

Citrinin analysis was carried out by the China National Research Institute of Food and Fermentation Industries, China Center of Industrial Culture Collection (CICC), according to GB 5009.222-2016, using immunoaffinity column cleanup combined with HPLC–FLD. Chromatographic separation was performed with methanol and 0.1% (*v*/*v*) aqueous phosphoric acid (70:30, *v*/*v*) as the mobile phase, and the injection volume was 50 μL. Fluorescence detection was performed at an excitation wavelength of 350 nm and an emission wavelength of 500 nm. The LOD and LOQ were 4 μg/kg and 12 μg/kg, respectively. The retention time of citrinin was confirmed with an external standard at approximately 8.97 min.

Definition of not detected (ND): ND was defined as the absence of a characteristic peak within the citrinin retention-time window with a response below the method LOD.

### 2.4. Genome Sequencing, Assembly, and Annotation

High-molecular-weight genomic DNA of BC20 was sequenced using a hybrid strategy combining PacBio long reads and Illumina short reads. Long-read sequencing was performed on the PacBio Sequel platform with 20 kb SMRTbell libraries, and short-read sequencing was conducted on the Illumina NovaSeq platform using the PE150 strategy with an insert size of approximately 350 bp. PacBio data were used for de novo assembly, followed by polishing with Illumina reads using SMRT Link v5.0.1. Assembly quality was assessed by total genome size, GC content, N50, and completeness.

Protein-coding genes were predicted using a combination of ab initio prediction with AUGUSTUS v2.7 [17] and homology-based prediction with GeneWise v2.4.1 [18], followed by integration with EVM [19]. For non-coding RNA annotation, tRNAs were predicted with tRNAscan-SE 2.0 [20] and rRNAs with RNAmmer v1.2 [21]. Other ncRNAs were identified by searching the Rfam database using cmsearch v1.1rc4 [22]. Functional annotation was performed against the COG, CAZy, and DFVF databases using DIAMOND [23] with thresholds of *e*-value ≤ 1 × 10^−5^, identity ≥ 40%, and coverage ≥ 40%.

Because eukaryotic 5.8S rRNA is highly conserved, short, and typically arranged in tandem with 18S and 28S rRNAs, automated annotation tools may omit it or merge it with adjacent sequences during large-fragment parsing. This is a known limitation of automated pipelines and does not indicate physical absence in the genome.

### 2.5. Comparative Genomics

Eighteen publicly available *Monascus* genome assemblies were retrieved from the GenBank database and analyzed together with BC20, giving a total of 19 genomes (Supplementary Table S2). Genome completeness was evaluated using BUSCO with the fungi_odb10 dataset [24]. Average nucleotide identity (ANI) was calculated using skani v0.2.1 [25], followed by hierarchical clustering based on ANI distances. A phylogenomic tree was inferred from single-copy orthologs identified by OrthoFinder and reconstructed using STAG [26], with *Aspergillus oryzae* RIB40 as the outgroup.

Whole-genome synteny was examined using assembly-to-assembly alignments generated with minimap2 (-x asm5) [27]. High-quality alignments were filtered and visualized with NGenomeSyn [28]. Gene families and single-copy orthologs were identified using OrthoFinder v2.5.x. Core secondary metabolite loci, including the citrinin and *mk* loci, were identified by homology searches using key anchor genes such as *ctnA* and *mkA*. Genomic coordinates and relative gene order were extracted from GFF3 annotations, and gene neighborhood architecture was visualized using the R package gggenes. For further sequence-level characterization of the BC20 citrinin-associated region, the citrinin biosynthetic gene cluster of *M. ruber* M7 (GenBank accession KT781075.1; MIBiG accession BGC0001338) was used as the reference and aligned to the homologous region of the BC20 genome assembly using minimap2. Sequence variants were called using paftools.js, normalized using bcftools, and functionally annotated using a custom SnpEff database constructed from the M7 BGC annotation. Candidate inverted-repeat (IR) elements within the BC20 citrinin-associated region (Contig8:1,240,000–1,310,000) were identified using EMBOSS inverted with a minimum score threshold of 50 and a maximum repeat span of 30 kb.

### 2.6. Antifungal Susceptibility Testing

Antifungal susceptibility testing was included as an additional component of strain characterization to establish the in vitro response profile of BC20 to major antifungal agents. For a viable filamentous fungus considered for food use, such information provides supplementary safety-related information and may serve as a reference for risk management in the event of opportunistic infection or accidental exposure. The antifungal susceptibility of BC20 to seven agents was determined using the broth microdilution method in accordance with CLSI M38 (2017). The tested agents were caspofungin, amphotericin B, itraconazole, ketoconazole, fluconazole, voriconazole, and 5-fluorocytosine. These agents represent major antifungal classes with different mechanisms of action, including echinocandins, polyenes, azoles, and pyrimidine analogues. The assay was performed in RPMI 1640 medium containing L-glutamine but without sodium bicarbonate and buffered to pH 7.0 with 0.165 M MOPS.

The inoculum was prepared by washing conidia from agar plates and adjusting the final concentration to 0.4–5 × 10^4^ CFU/mL. Antifungal agents were prepared as two-fold serial dilutions in 96-well microtiter plates. After inoculation, the incubation conditions were adjusted to 30 °C for 72 h to better match the growth characteristics of *Monascus* spp. Endpoint interpretation followed CLSI guidelines. For amphotericin B and most azoles, the MIC was defined as the lowest concentration showing complete growth inhibition. For fluconazole, ketoconazole, and 5-fluorocytosine, the MIC was defined as the lowest concentration causing approximately 50% growth inhibition. For caspofungin, the minimum effective concentration (MEC) was defined as the lowest concentration causing microscopically visible alterations in hyphal morphology.

### 2.7. Statistical Analysis

Statistical analyses and data visualization were performed using GraphPad Prism and R.

### 2.8. Data Availability

The whole-genome assembly and raw sequencing data of BC20 have been deposited in the NCBI database under BioProject accession number PRJNA1004441. Details of the comparative genomic analysis are provided in Supplementary Table S2.

## 3. Results

### 3.1. Citrinin Was Not Detected Across Eight Cultivation Systems

**Figure 1:**
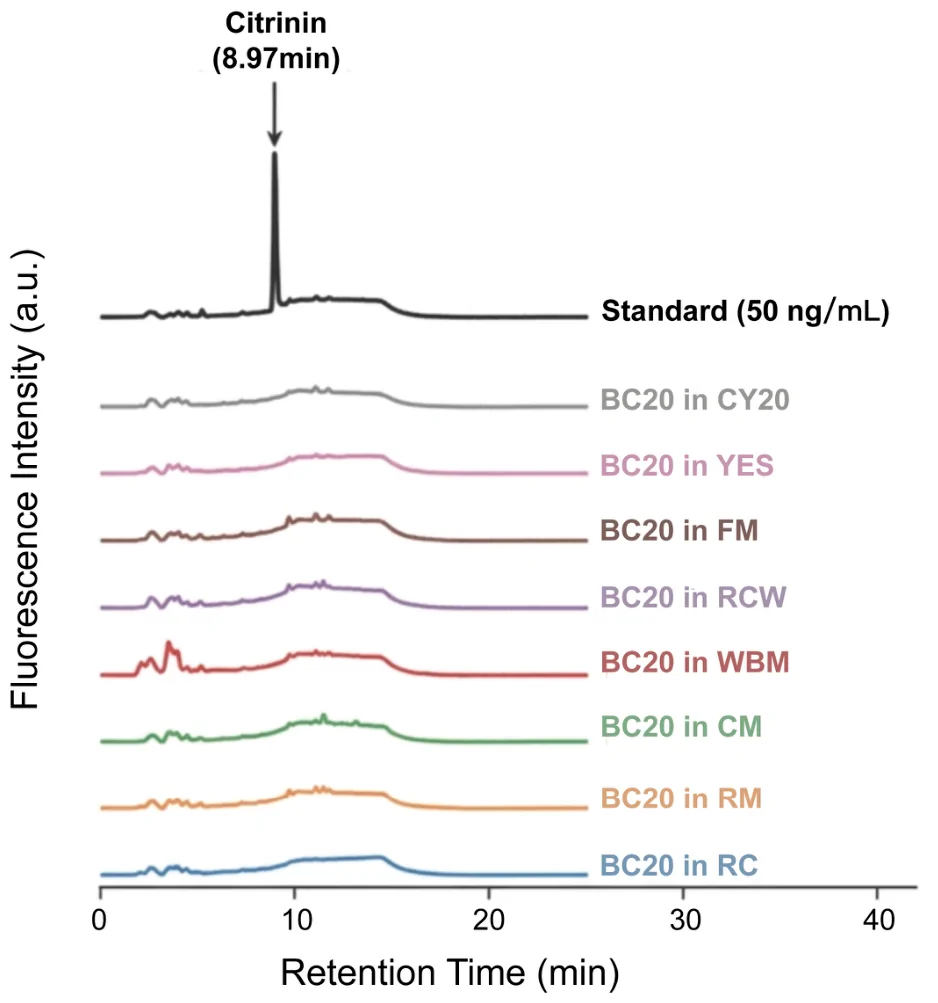
Citrinin screening of BC20 in eight cultivation systems. HPLC–FLD chromatograms of the citrinin standard and representative BC20 fermentation extracts. The citrinin standard eluted at approximately 8.97 min, whereas no corresponding peak was detected in BC20 samples.

To characterize the basic physicochemical conditions of the different cultivation systems, the initial pH values of the eight systems ranged from 5.50 to 6.80, while the pH values after 14 days of cultivation ranged from 4.46 ± 0.11 to 6.94 ± 0.06, indicating matrix-dependent pH changes during cultivation. The initial water activity (aw) values of the four solid substrates ranged from 0.970 ± 0.007 to 0.992 ± 0.002 (Supplementary Table S3). These measurements defined the physicochemical conditions under which BC20 was cultivated and provided the basis for further evaluation of its citrinin phenotype across food-relevant environments. The stability of the low-citrinin-risk phenotype of BC20 was first examined across diverse food-relevant matrices. HPLC–FLD analysis showed a clear peak for the citrinin standard at a retention time of 8.97 min. In contrast, extracts from all BC20F fermentation samples showed no characteristic peak within the corresponding retention-time window (Figure 1). Consistent with this observation, citrinin was not detected under all tested conditions. These conditions included standard fungal media, a dairy-based liquid matrix, and solid substrates containing cereals and bran. The consistent ND phenotype across these matrices prompted us to examine its genetic basis.

### 3.2. Species Identification and Chromosome-Scale Genome Assembly of BC20

**Figure 2:**
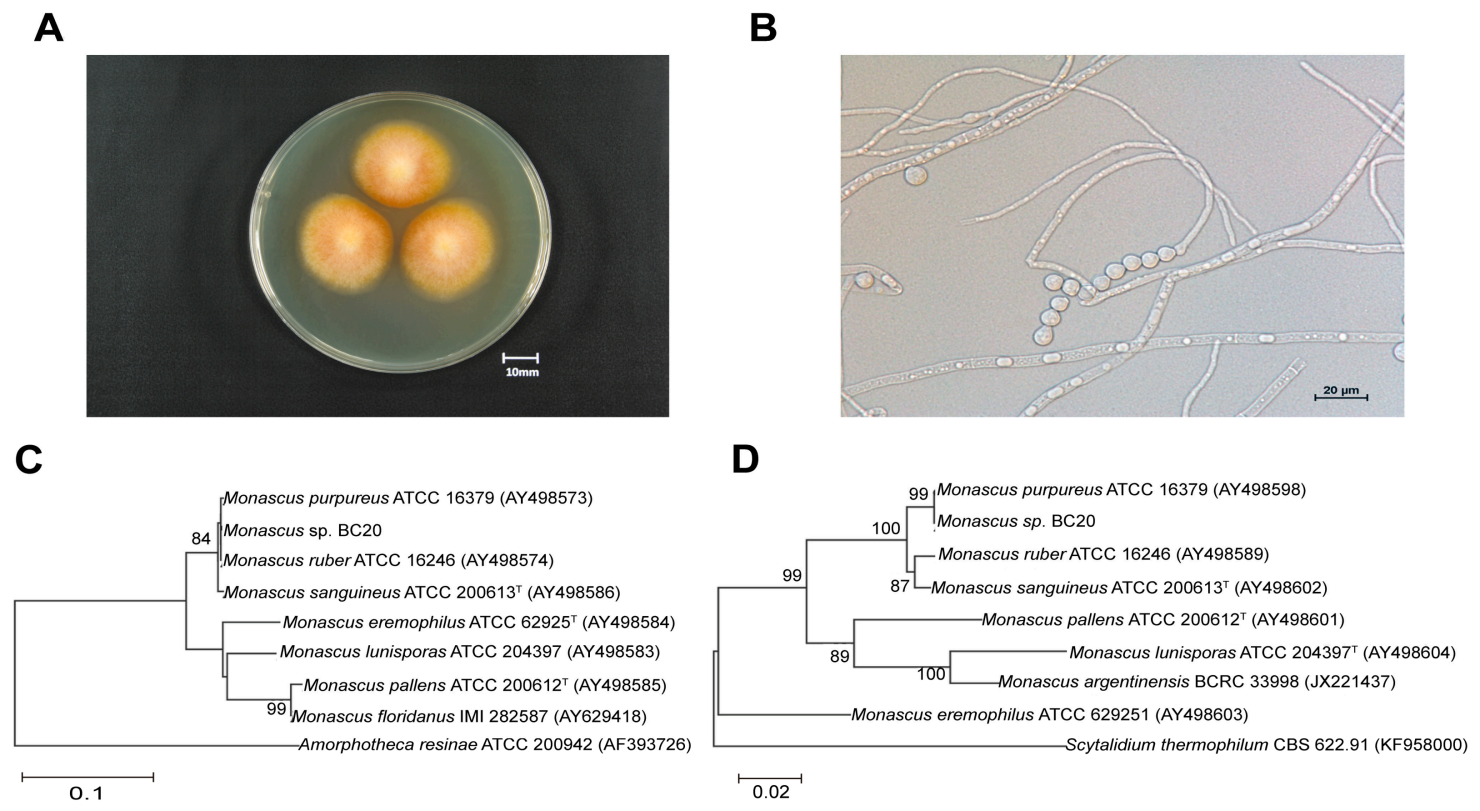
Morphology and molecular identification of BC20. (**A**) Colony morphology of BC20 on MEA after incubation at 30 °C for 7 d. (**B**) Microscopic morphology of BC20. (**C**) Neighbor-joining phylogenetic tree based on ITS sequences. (**D**) Neighbor-joining phylogenetic tree based on *BenA* sequences.

After 7 days of growth on MEA, BC20 formed orange-red velvety colonies with relatively regular margins. The reverse side showed stronger pigmentation with slight diffusion of soluble pigments (Figure 2A). Microscopic examination revealed septate hyphae and subglobose to ovoid conidia (Figure 2B). Phylogenetic analyses based on ITS and *BenA* sequences consistently placed BC20 within the *Monascus ruber* lineage (Figure 2C,D).

Using a hybrid assembly strategy based on PacBio long reads and Illumina short reads, we generated a chromosome-scale genome assembly for BC20 (Supplementary Figure S1). The genome size was 26,189,058 bp with a GC content of 48.85%. LINE elements accounted for 3.5% of the assembled genome. A total of 6720 protein-coding genes were predicted, together with 162 tRNAs and 31 rRNAs (Table 1). This high-contiguity assembly enabled reliable synteny analysis and locus-level comparison and reduced the chance of mistaking assembly fragmentation for biological deletion.

### 3.3. Comparative Genomics Places BC20 Within the Ruber–Pilosus Clade

**Figure 3:**
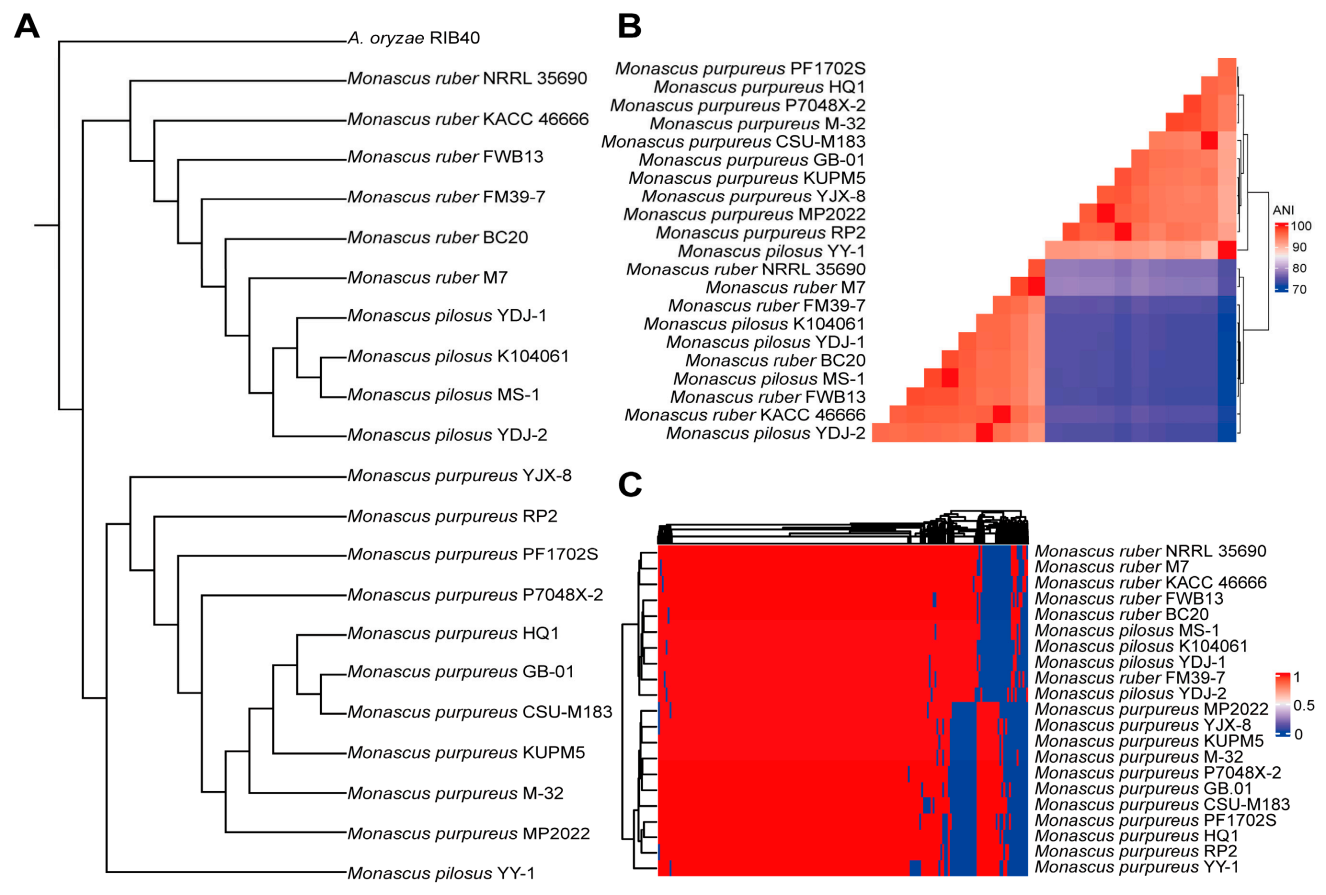
Comparative genomic analysis of BC20 and 18 publicly available *Monascus* genomes. (**A**) Phylogenomic tree inferred from single-copy orthologs. (**B**) ANI heatmap showing genomic relatedness among the 19 analyzed genomes. ANI, average nucleotide identity. (**C**) Orthogroup distribution among the analyzed genomes.

To place BC20 in an evolutionary context, it was compared with 18 publicly available *Monascus* genomes. BUSCO analysis using the fungi_odb10 dataset showed 99.3% complete BUSCOs, including 98.4% single-copy and 0.9% duplicated BUSCOs, with 0.3% fragmented and 0.4% missing BUSCOs (Supplementary Figure S2). Both ANI clustering and phylogenomic reconstruction consistently placed BC20 within the *ruber*–*pilosus* clade (Figure 3A,B). The ANI heatmap showed clear separation within the genus, with *M. purpureus* forming an independent group and *M. ruber* and *M. pilosus* forming a closely related lineage that included BC20 (Figure 3B).

A phylogenomic tree based on single-copy orthologous proteins showed the same overall topology. The *M.* *purpureus* clade was clearly separated from the *ruber–pilosus* clade, and BC20 clustered closely with reference *M. ruber* genomes and adjacent to *M. pilosus* (Figure 3A). This phylogenetic placement helped interpret locus-level variation in BC20.

### 3.4. Conserved Genome Backbone Supports Targeted Comparison of Risk Loci

**Figure 4:**
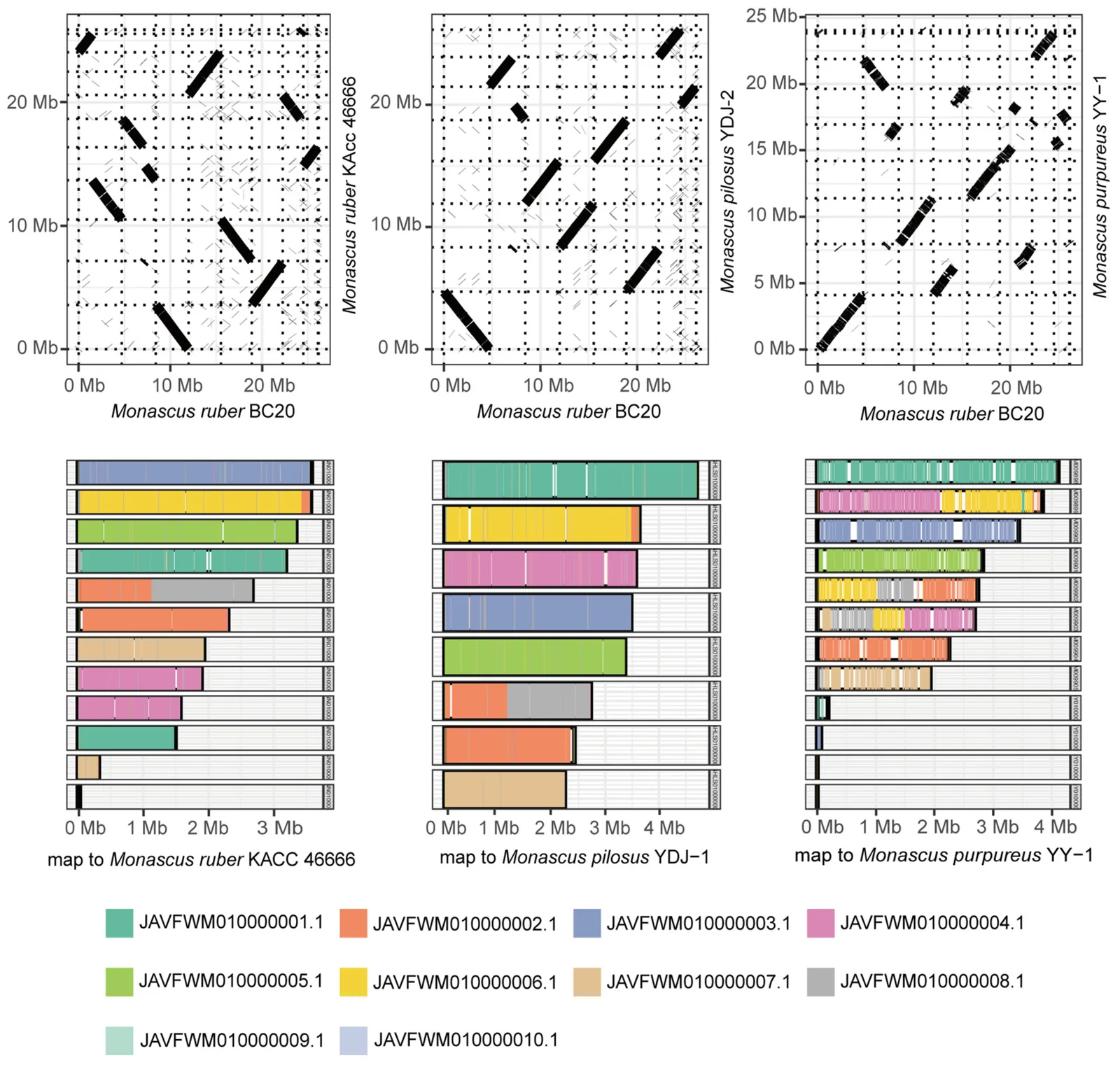
Whole-genome synteny analysis of BC20 and representative *Monascus* genomes. Genome-wide collinearity between BC20 and representative genomes from the *ruber–pilosus* and purpureus groups. BC20 showed extensive linear synteny with representative *ruber–pilosus* genomes. Black diagonal segments represent syntenic regions between BC20 and the corresponding reference genome.

BC20 was further compared with representative genomes from the ruber and pilosus groups at the whole-genome level. Extensive linear synteny was observed between BC20 and the reference *M. ruber* genome (Figure 4), indicating broad conservation of genome organization within this lineage. Pan-genome and orthogroup analyses further showed that the analyzed genomes shared a large core orthogroup set, whereas most variation was confined to a smaller accessory component (Figure 3C; Supplementary Figure S4). These results show that BC20 is part of a relatively conserved genomic background, making variation at candidate risk loci easier to interpret.

### 3.5. The Citrinin-Associated Locus Shows a Remnant Architecture in BC20, with Similar Patterns Observed in Related Genomes of the Ruber–Pilosus Clade

**Figure 5:**
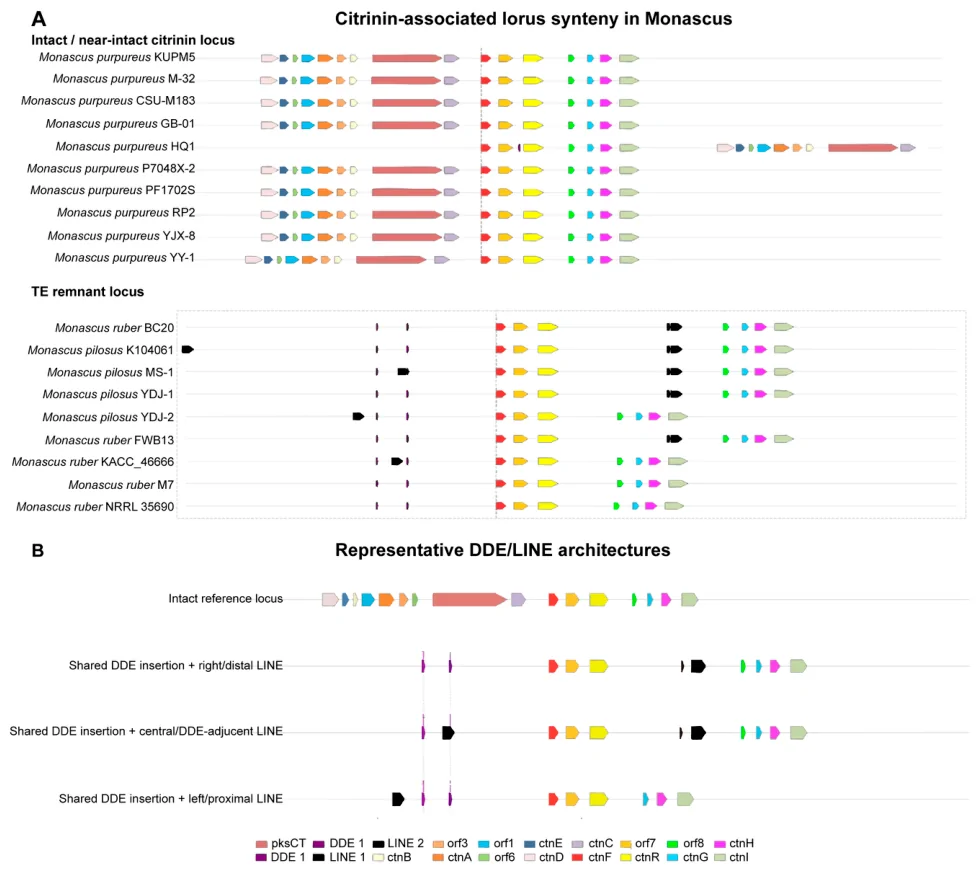
Comparative organization of the citrinin-associated locus in BC20 and related *Monascus* genomes. (**A**) Global synteny comparison of the citrinin-associated locus across representative *Monascus* strains, showing intact or near-intact clusters in most *M. purpureus* genomes and a remnant-locus architecture in BC20 and related ruber–pilosus genomes. (**B**) Representative local comparison of remnant-locus configurations. A shared DDE-associated breakpoint was observed, whereas LINE-like elements occurred at variable strain-dependent positions within the disturbed syntenic region.

The genomic status of the citrinin-associated locus was then examined. Synteny analysis showed that the *M. purpureus* reference genome retained a relatively intact citrinin biosynthetic region (Figure 5A), including the core biosynthetic gene *pksCT* and adjacent tailoring and regulatory genes. In contrast, the corresponding syntenic region in BC20 no longer preserved an intact citrinin cluster configuration and instead exhibited a clear remnant architecture, with partial loss or disruption of the citrinin-associated region (Figure 5A). Similar patterns were also observed in several related genomes from the *ruber–pilosus* clade, indicating that the structural pattern seen in BC20 was not an isolated case. This locus configuration was consistent with the non-detectable citrinin phenotype of BC20 across the tested matrices and provided a structural explanation for that phenotype.

Further comparison suggested that these remnant loci were not the result of random degradation. Instead, BC20 and the related genomes shared a similar disrupted backbone, characterized by DDE-related insertions at comparable syntenic positions, whereas LINE-like elements showed variable positional patterns among strains (Figure 5B). These differences gave rise to several secondary remnant configurations, but all remained clearly distinct from the intact locus observed in most *M. purpureus* genomes. In BC20 and the related genomes, annotations associated with transposable elements, including DDE transposases and LINE-like elements, were also found near the disrupted syntenic region (Figure 5). Further sequence-level analysis of the retained coding regions in BC20 detected no variants annotated as start_lost, stop_gained, or stop_lost. In addition, three candidate inverted-repeat (IR) elements were identified within the BC20 citrinin-associated region (Supplementary Table S4). Taken together, these observations suggest an association between degeneration of this locus and nearby TE-rich regions. However, this association should not be taken as direct mechanistic evidence. At this stage, these regions are better regarded as candidate targets for future breakpoint-level validation.

### 3.6. The Monacolin K Locus Is Syntenically Conserved in BC20

To exclude the possibility that the apparent absence of the citrinin-associated region in BC20 resulted from assembly fragmentation or broad degradation of secondary metabolite loci, we examined the monacolin K (*mk*) biosynthetic locus as an internal control. Previous studies have reported variation in the *mk* cluster among different *Monascus* strains [11], making it a useful locus for evaluating both assembly fidelity and retention of fermentation-related biosynthetic potential.

Comparative analysis of the *mk* region showed that the core *mk* genes and their surrounding genomic organization were clearly identifiable in BC20 (Supplementary Figure S4). The cluster contained major core genes, including *mkA*, *mkC*, *mkD*, *mkE*, *mkF*, *mkG*, and *mkH*, and displayed synteny similar to that of several *M. ruber* genomes. Unlike the citrinin-associated region, no major deletion or strong disruption signal was observed in this locus. These results suggest that the citrinin-negative phenotype of BC20 is associated with locus-specific deletion or disruption rather than assembly-derived pseudo-absence.

### 3.7. Functional Annotation Suggests Carbohydrate Utilization Potential

**Figure 6:**
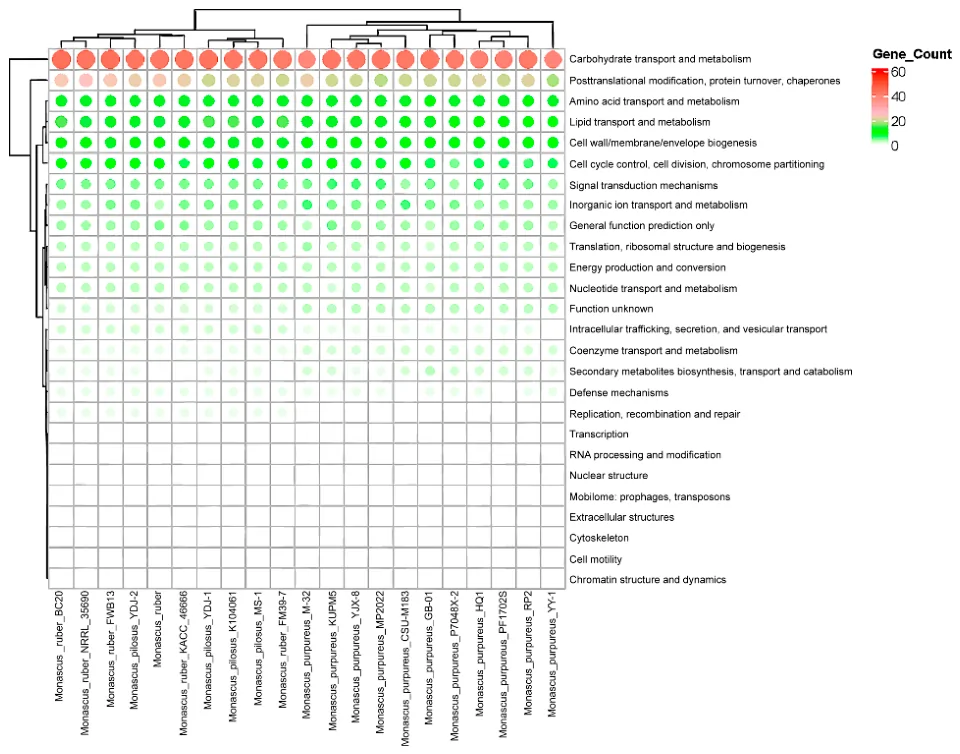
COG functional classification of BC20 and representative *Monascus* genomes. Distribution of genes assigned to major COG functional categories in BC20 and representative *Monascus* genomes. COG, Clusters of Orthologous Groups.

**Figure 7:**
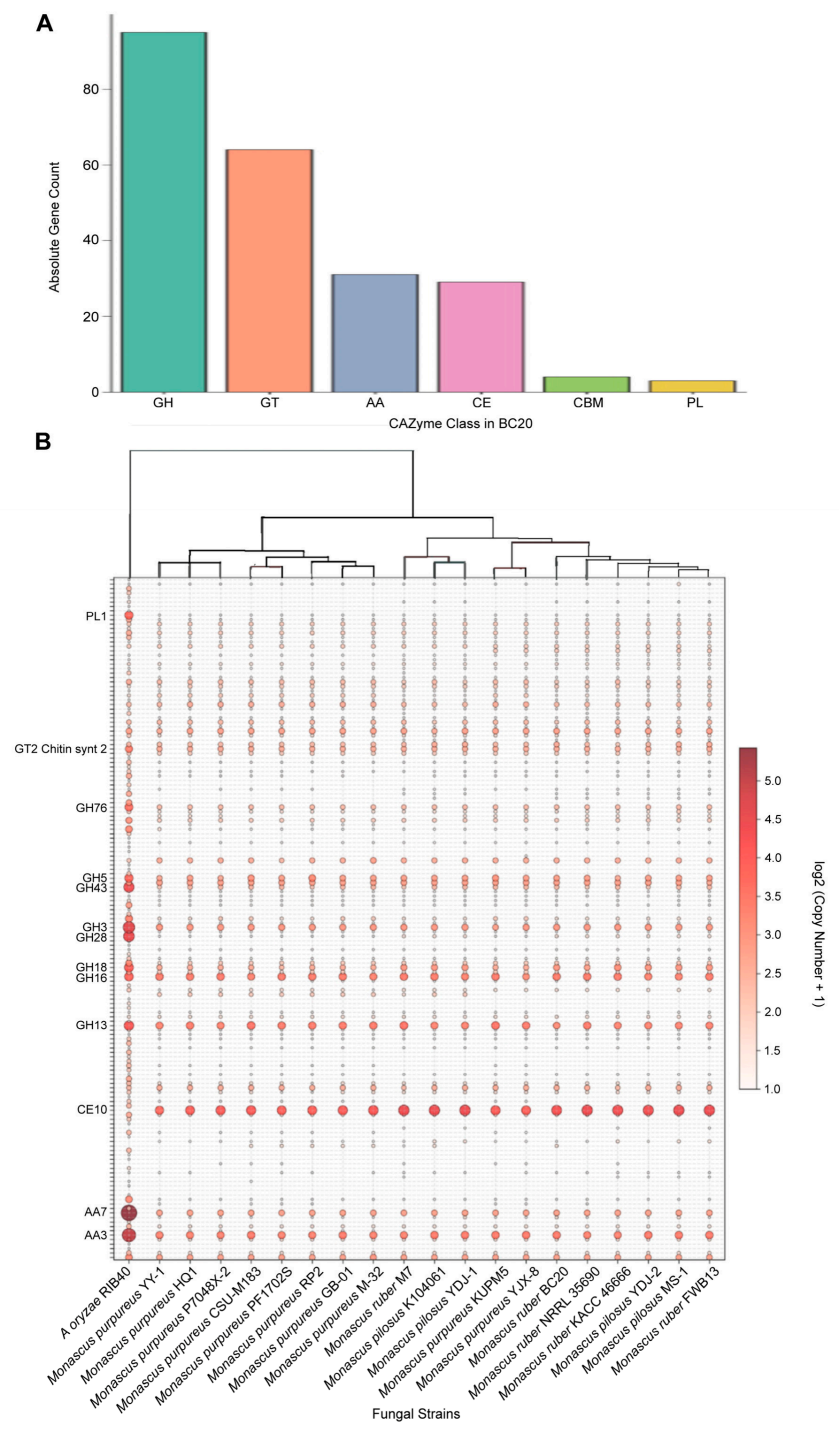
Functional annotation of BC20. (**A**) Distribution of CAZyme classes in BC20 and related genomes. (**B**) Heatmap of selected CAZyme families associated with carbohydrate utilization.

**Figure 8:**
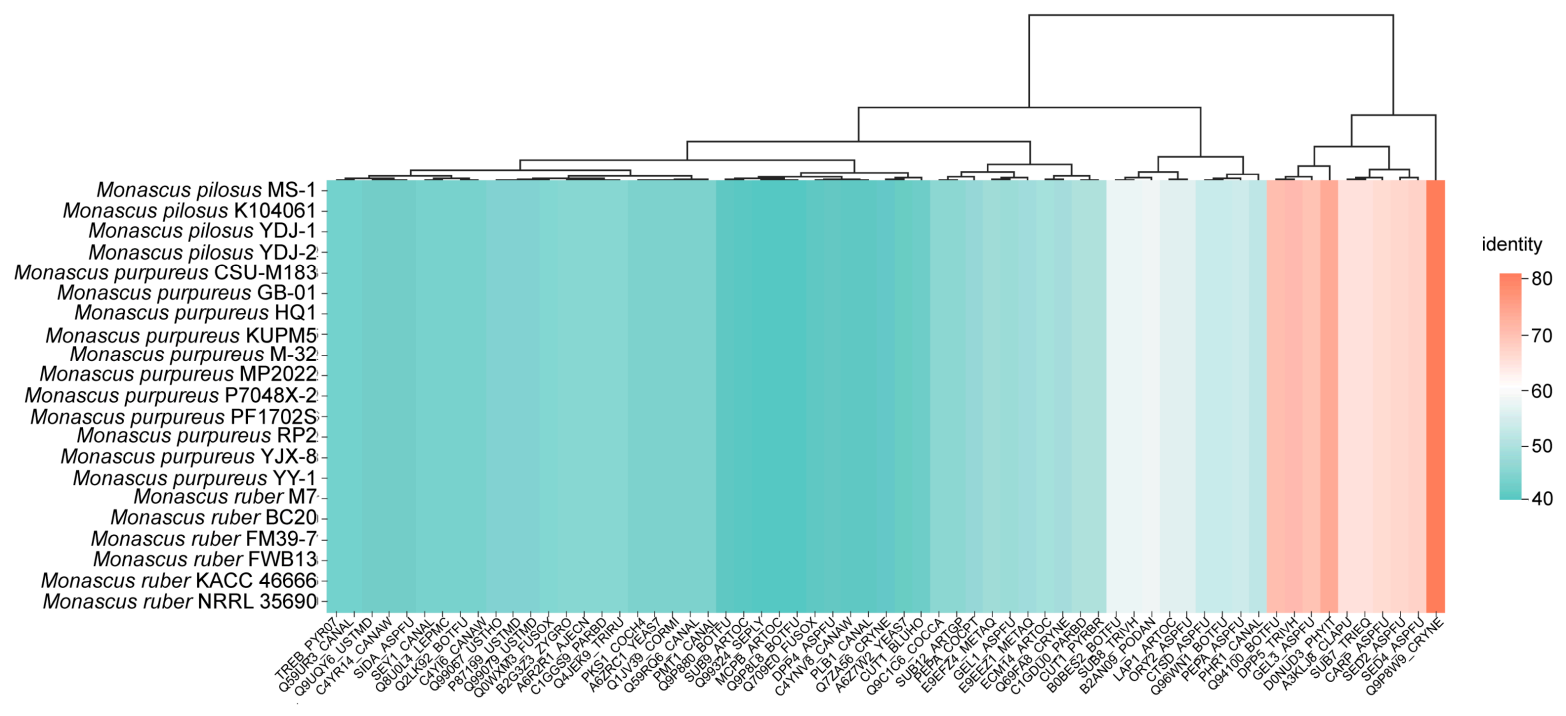
Heatmap of predicted secreted protein homologs in BC20 and representative *Monascus* genomes. Colors indicate sequence identity (%) of predicted secreted protein homologs across the analyzed genomes.

After establishing the relationship between the low-citrinin-risk phenotype and the locus state, we evaluated the genomic potential of BC20 for fermentation-related functions. COG classification showed a broadly similar functional profile between BC20 and other *Monascus* genomes, with genes involved in carbohydrate transport and metabolism well represented (Figure 6). CAZyme analysis showed that glycoside hydrolases (GH) and glycosyltransferases (GT) were the dominant classes in BC20 (Figure 7), while secretome analysis indicated broad conservation of predicted secreted proteins across the genus (Figure 8). These results provide complementary genomic information on the potential of BC20 for substrate utilization.

### 3.8. Antifungal Susceptibility Profile

MIC testing based on CLSI M38 (2017) showed the following values for BC20 (Table 2): caspofungin MEC, 0.0156 μg/mL; amphotericin B MIC, 0.5 μg/mL; itraconazole MIC, 2 μg/mL; ketoconazole MIC, 8 μg/mL; fluconazole MIC, 32 μg/mL; voriconazole MIC, >32 μg/mL; and 5-fluorocytosine MIC, 16 μg/mL. These results provide a quantitative profile of the in vitro antifungal responses of BC20 to the tested agents.

## 4. Discussion

*Monascus* species have important applications in food fermentation, but the persistent risk of citrinin contamination limits their wider use in food systems [29], particularly in applications such as dairy-related matrices where safety expectations are high. For candidate strain selection, the key issue is not only whether citrinin is absent in a single test but also whether this phenotype remains stable under food-relevant conditions and whether it can be explained by traceable genetic evidence. In the present study, we evaluated the furu-derived strain *Monascus ruber* BC20 by integrating phenotypic data, genome analysis, and additional strain-characterization data.

BC20 consistently showed a non-detectable citrinin phenotype across eight different culture systems, including fungal media, dairy-based systems, and solid cereal- and bran-based substrates. Because these matrices differ substantially in nutrient composition, moisture conditions, and oxygen transfer, the consistency of the non-detectable phenotype across them provides useful support for the stability of this trait under food-relevant conditions. As all matrices were sterilized and inoculated with the same standardized inoculum, these results reflect citrinin production under controlled monoculture conditions rather than the competitive establishment of BC20. The effect of inoculum size itself could not be assessed because no inoculum gradient or quantitative growth measurements were included.

The chromosome-level genome assembly further allowed this phenotype to be interpreted within a reliable genomic framework. For biosynthetic loci, which are often located in structurally variable regions, assembly continuity is critical for distinguishing true locus alteration from assembly fragmentation. The high-quality assembly of BC20 therefore provided an appropriate basis for synteny analysis and locus-level comparison.

BC20 was also interpreted within an intra-genus comparative framework rather than as an isolated genome. ANI analysis, phylogenomic inference, and whole-genome synteny consistently placed BC20 within the *ruber–pilosus* clade, which showed a relatively conserved genomic backbone. This comparative context allowed variation in the citrinin-associated region of BC20 to be interpreted against a stable genomic background rather than as a strain-specific anomaly.

Within this framework, the citrinin-associated locus in BC20 showed structural features consistent with its phenotype. Most *M. purpureus* genomes retained a relatively intact citrinin-associated region, whereas the corresponding syntenic region in BC20 no longer preserved the intact configuration and instead showed a clear remnant architecture. Similar patterns were also observed in several related genomes from the *ruber–pilosus* clade, indicating that the BC20 structure was not an isolated case. The shared disrupted backbone and DDE-related insertions at comparable syntenic positions suggest that these remnant loci may reflect a lineage-associated history of structural remodeling of the citrinin-associated region in the *ruber–pilosus* clade. In contrast, the variable positions of LINE-like elements among strains are consistent with subsequent strain-specific rearrangements.

Transposable element-related annotations, including DDE transposases and LINE-like elements, were detected near the disturbed region in BC20. Their occurrence near the disrupted citrinin-associated region suggests that historical structural rearrangements may have contributed to the formation of the remnant locus. From the perspective of phenotype stability, the resulting disrupted locus architecture provides a more stable structural basis for the citrinin-non-detectable phenotype than an intact biosynthetic cluster that is only conditionally silenced. At present, however, the evidence is derived mainly from comparative genome structure and co-localization. These observations are therefore better interpreted as structural association rather than direct proof of mechanism. Further confirmation will require breakpoint-spanning reads, targeted PCR, and additional molecular validation.

To reduce the possibility of pseudo-absence, we further examined the mk locus as an internal control. In BC20, the mk region remained structurally recognizable and syntenically conserved. This does not support the idea that the citrinin-associated locus was apparently absent merely because of poor assembly quality or broader collapse of secondary metabolite-rich regions. Instead, it is more consistent with a locus-specific structural disturbance in BC20.

Beyond the citrinin-associated locus, functional annotation indicated that BC20 retained genes potentially relevant to fermentation. COG classification and CAZyme analysis showed the presence of functions associated with carbohydrate utilization and substrate hydrolysis, while the predicted secretome was broadly conserved among the analyzed *Monascus* genomes. These genomic features suggest that BC20 may retain traits relevant to substrate utilization, although their contribution to fermentation performance remains to be experimentally determined. Further studies should therefore fermentation performance, growth capacity, examine pigment production, volatile formation, hydrolytic activity, and sensory properties directly in target food matrices.

Given that BC20 was isolated from furu, its relevance is particularly linked to traditional Chinese fermented foods [30]. *Monascus* is an important fungal component during furu post-fermentation, and naturally occurring strains recovered from this environment represent a relevant resource for strain selection. The disrupted architecture of the citrinin-associated locus identified in BC20 further highlights the value of evaluating naturally occurring *Monascus* resources from traditional fermentation systems at both phenotypic and genomic levels. BC20 and related isolates therefore warrant further investigation for their potential use in traditional Chinese fermented foods.

The present results also indicate how phenotypic and genomic information can be combined during strain screening. Citrinin production can first be examined under multiple food-relevant conditions, after which strains showing consistently undetectable citrinin can be subjected to locus-level genomic analysis. This approach allows strains with a structurally disrupted citrinin-associated region to be distinguished from those retaining an intact locus and provides an additional basis for selecting candidates for subsequent fermentation studies.

Citrinin was not detected in any of the eight cultivation systems examined in this study. However, differences in matrix composition may influence extraction efficiency and analyte recovery, particularly in protein-rich dairy matrices and starch-rich cereal substrates. Matrix-specific validation, including spike-recovery and matrix-effect assessment, will therefore be required in further analytical work. The agreement between the phenotypic results obtained across the tested systems and the remnant architecture of the citrinin-associated locus supports further evaluation of BC20 as a candidate strain for food fermentation.

## 5. Conclusions

This study identified a locus-specific structural disruption of the citrinin-associated region in *Monascus ruber* BC20. Citrinin was not detected after 14 days of cultivation across eight media and food-related matrices, consistent with the remnant architecture of the citrinin-associated locus revealed by chromosome-scale comparative genomic analysis. In contrast, the *mk* locus remained structurally conserved, further supporting the locus-specific nature of this genomic alteration. Together, the concordance between the phenotypic and genomic evidence supports BC20 as a candidate *M. ruber* strain with undetectable citrinin for further evaluation in cereal-based fermented foods and dairy fermentation systems. Given the current lack of matrix-specific analytical validation, further development should focus on spike-recovery and matrix-effect validation, expanded safety evaluation, and pilot-scale fermentation trials.

## Figures and Tables

**Table 1:** General features of the genome sequences of strain BC20.

General Features	BC20
Genome size (bp)	26,189,058
Gene number	6720
Gene total length (bp)	10,131,042
Gene total length/genome (%)	38.68
Gene average length (bp)	1508
GC content (%)	48.85%
LINE elements (% of genome)	3.5
tRNA	162
5S (de novo)	24
18S (de novo)	3
28S (de novo)	4
sRNA	2
snRNA	15

**Table 2:** Antifungal susceptibility profile of BC20 determined by the CLSI M38 broth microdilution method.

Antifungal Agent	Endpoint	Value (μg/mL)
Itraconazole	MIC	2
Ketoconazole	MIC	8
Voriconazole	MIC	>32
Caspofungin	MEC	0.0156
5-fluorocytosine	MIC	16
Fluconazole	MIC	32
Amphotericin B	MIC	0.5

## Data Availability

The datasets generated in this study are available in public repositories. The whole-genome assembly and raw sequencing data of BC20 have been deposited in the NCBI database under BioProject accession number PRJNA1004441.

## Supplementary Materials

The following supporting information can be downloaded at https://www.mdpi.com/article/10.3390/foods15173091/s1: Table S1: Composition, sterilization, and incubation conditions of the eight cultivation systems used for BC20; Table S2: Publicly available *Monascus* genomes used for comparative analysis with BC20; Table S3: Initial and post-fermentation pH of the eight cultivation systems and initial water activity (aw) of the solid substrates used for BC20; Table S4: Candidate inverted repeats detected in the BC20 citrinin-associated region; Figure S1: Circular overview of the chromosome-scale genome assembly of BC20; Figure S2: BUSCO assessment of the BC20 genome assembly using the fungi_odb10 dataset; Figure S3: Comparative genomics and whole-genome phylogenomics; Figure S4: Comparative analysis of the monacolin K (mk) biosynthetic locus in representative *Monascus* genomes.
