# Supplementary material for: Chromosome-Scale Genome Analysis Reveals Locus-Specific Disruption of the Citrinin-Associated Region in a Furu-Derived Monascus ruber Strain BC20
Source: Foods. 2026 Aug 31;15(17):3091. doi: 10.3390/foods15173091 (PMC13565668; doi:10.3390/foods15173091)
Supplement: Supplementary file 1 [file foods-15-03091-s001.zip › Supplementary_Materials clean.pdf]

Supplementary Materials: Supplementary Tables S1–S4 and Supplementary Figures S1–S4.

Supplementary Table S1. Composition, sterilization, and incubation conditions of the eight cultivation systems used for BC20.

Code	System type	Composition / preparation	Sterilization	Incubation
CY20 (Czapek yeast extract sucrose medium)	Liquid	K_2HPO_4, 1 g; Czapek concentrate, 10 mL; yeast extract, 5 g; sucrose, 50 g; distilled water to 1000 mL	Autoclaved at 121 °C for 15 min	30 °C, 120 rpm, 14 d
RC (Rice flour–corn steep liquor medium)	Liquid	Rice flour, 5 g; corn steep liquor, 4 g; $ZnSO_4·7H_2O$, 0.001 g; $CuSO_4·5H_2O$, 0.05 g; distilled water to 1000 mL	Autoclaved at 121 °C for 15 min	30 °C, 120 rpm, 14 d
YES (Yeast extract sucrose medium)	Liquid	Yeast extract, 10 g; sucrose, 50 g; distilled water to 1000 mL	Autoclaved at 121 °C for 15 min	30 °C, 120 rpm, 14 d
FM (Sterilized milk medium)	Liquid	Commercial sterilized milk (Bright Dairy), directly dispensed into culture vessels	Commercial sterilization at 137 °C for 4 s	30 °C, 120 rpm, 14 d
RM (Rice substrate)	Solid	Rice, 20 g, placed in a 1000 mL Erlenmeyer flask	Autoclaved at 121 °C for 20 min once daily for 3 consecutive days; cooled and loosened after each sterilization.	30 °C, 14 d

Code	System type	Composition / preparation	Sterilization	Incubation
CM (Corn substrate)	Solid	Ground corn grits, 20 g (particle size approximately 0.2–0.5 mm), placed in a 1000 mL Erlenmeyer flask	Same as RM	30 °C, 14 d
WBM (Wheat bran substrate)	Solid	Wheat bran, 20 g, placed in a 1000 mL Erlenmeyer flask	Same as RM	30 °C, 14 d
RCW (Rice–corn–wheat bran mix)	Solid	Rice, corn grits, and wheat bran mixed at a ratio of 1:1:1; 20 g of the mixture placed in a 1000 mL Erlenmeyer flask	Same as RM	30 °C, 14 d

Supplementary Table S2. Publicly available *Monascus* genomes used for comparative analysis with BC20.

No.	Accession Number	Strains
1	GCA_900184055.1	*Monascus ruber* M7
2	GCA_024449045.1	*Monascus ruber* KACC_46666
3	GCA_002976275.1	*Monascus ruber* FWB13
4	GCA_018806895.1	*Monascus pilosus* K104061
5	GCA_018806995.1	*Monascus pilosus* MS-1
6	GCA_018806905.1	*Monascus pilosus* YDJ-1
7	GCA_027569325.1	*Monascus ruber* NRRL_35690
8	GCA_018806955.1	*Monascus pilosus* YDJ-2
9	GCA_023624895.1	*Monascus purpureus* P7048X-2
10	GCA_011319195.1	*Monascus purpureus* YJX-8
11	GCA_004359145.1	*Monascus purpureus* GB-01
12	GCA_025999795.1	*Monascus purpureus* KUPM5
13	GCA_006542485.1	*Monascus purpureus* HQ1
14	GCA_023624875.1	*Monascus purpureus* PF1702S
15	GCA_003184285.1	*Monascus purpureus* YY-1
16	GCA_023935125.1	*Monascus purpureus* RP2
17	GCA_027497415.1	*Monascus purpureus* M-32
18	GCA_019320005.1	*Monascus purpureus* CSU-M183

Supplementary Table S3. Initial and post-fermentation pH of the eight cultivation systems and initial water activity (aw) of the solid substrates used for BC20.

Cultivation system	Initial pH	Final pH, 14 d	Initial (a_w)
YES	5.50	6.94 ± 0.06	
CY20	5.50	6.23 ± 0.12	
RC	5.50	6.25 ± 0.09	
FM	6.80	6.55 ± 0.07	
RM	6.70	4.70 ± 0.15	0.970 ± 0.007
CM	6.38	4.46 ± 0.11	0.981 ± 0.002
WBM	6.07	6.09 ± 0.12	0.977 ± 0.003
RCW	6.11	5.65 ± 0.10	0.992 ± 0.002

Supplementary Table S4. Candidate inverted repeats detected in the BC20 citrinin-associated region

IR	einverted score	Arm length	Matches	Identity	Gaps	BC20 Contig8 coordinates	Intervening sequence
IR1	77	28 bp	27/28	96%	0	1,244,309–1,244,336 and 1,264,130–1,264,157	19,793 bp
IR2	97	44 bp	39/44	88%	0	1,275,099–1,275,142 and 1,282,428–1,282,471	7,285 bp

IR	einverted score	Arm length	Matches	Identity	Gaps	BC20 Contig8 coordinates	Intervening sequence
IR3	60	34 bp	28/34	82%	0	1,283,896–1,283,929 and 1,286,081–1,286,114	2,151 bp

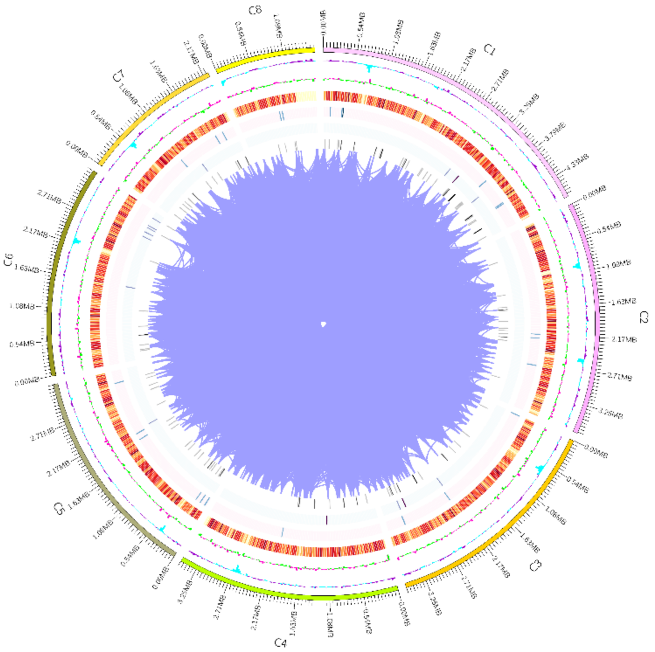

Supplementary Fig. S1. Circular overview of the chromosome-scale genome assembly of BC20. The eight chromosomes and major genomic features are shown, with inner links indicating intra-genomic homologous regions.

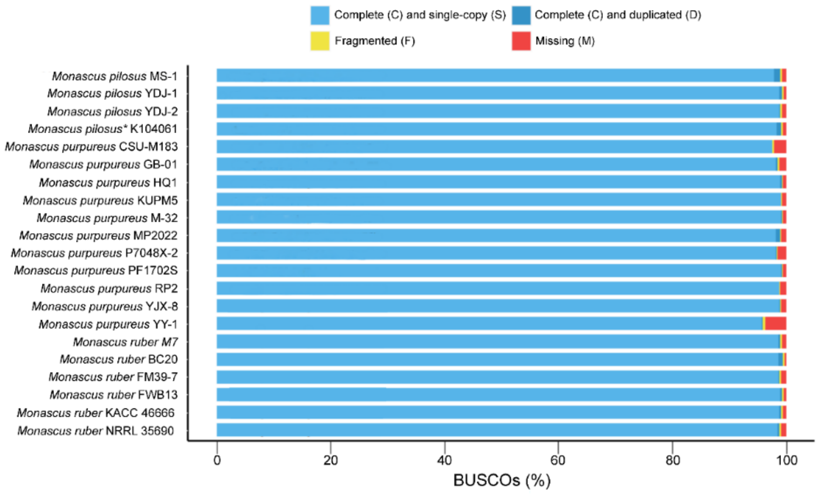

Supplementary Fig. S2. BUSCO assessment of the BC20 genome assembly using the fungi_odb10 dataset.

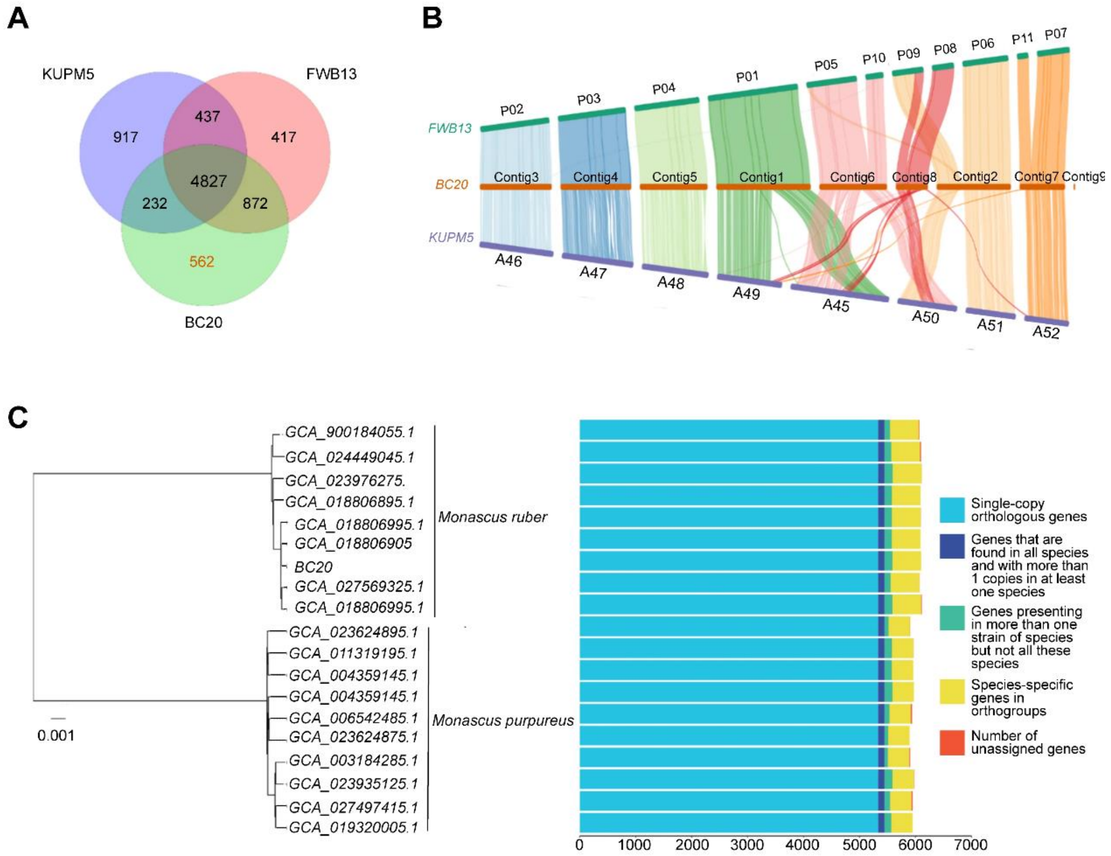

Supplementary Fig. S3. Comparative genomics and whole-genome phylogenomics. (A) Pan-genome Venn diagram of BC20, FWB13, and KUPM5. (B) Whole-genome collinearity of BC20 with *M. ruber* FWB13 and *M. purpureus* KUPM5. (C) Genome-wide phylogenomic tree and ortholog classification of representative *Monascus* genomes.

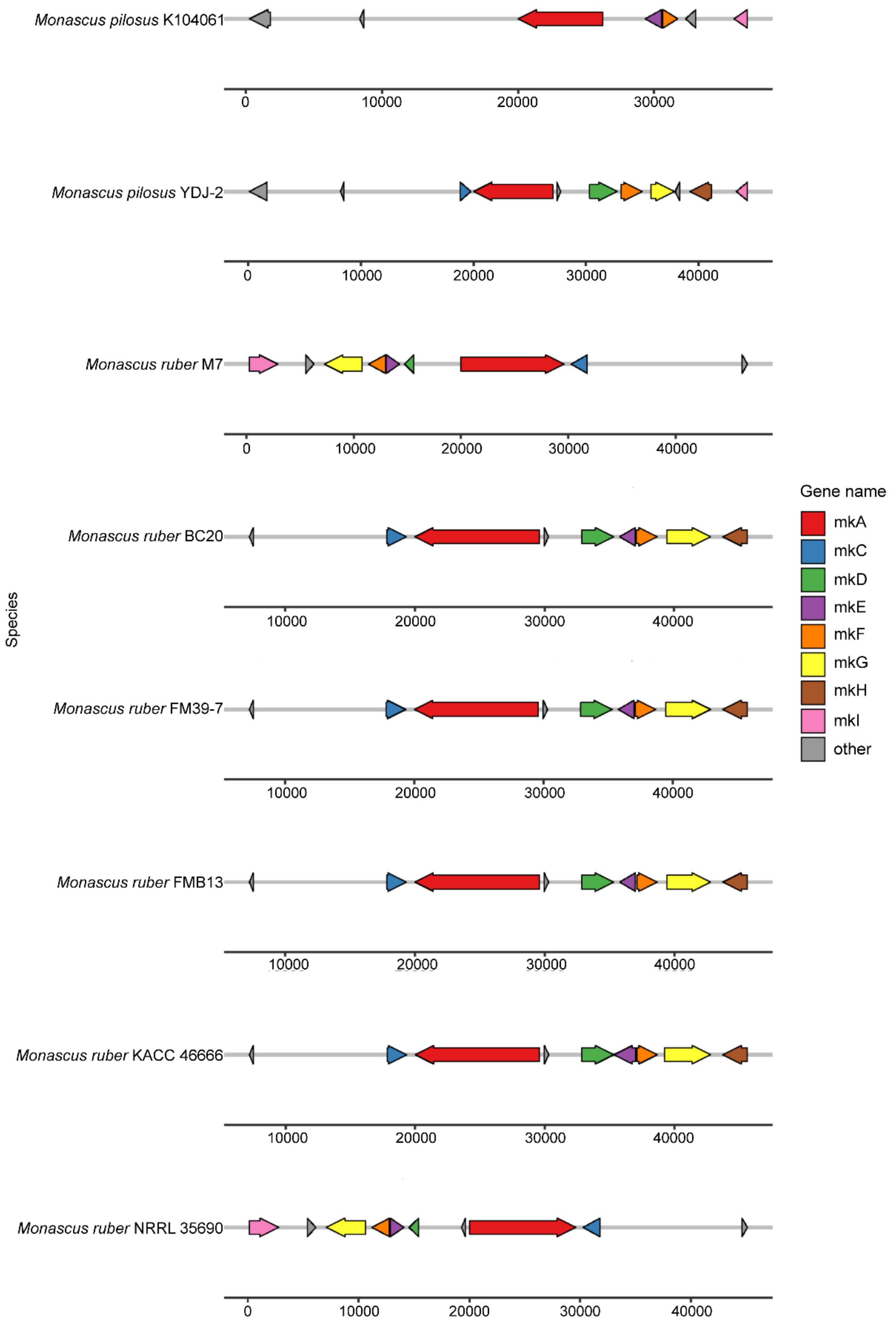

Supplementary Fig. S4. Comparative analysis of the monacolin K (*mk*) biosynthetic locus in representative *Monascus* genomes. The *mk* locus and

flanking regions are compared among BC20 and representative ruber–pilosus genomes.
